# Aberrant DNA Methylation of ABC Transporters in Cancer

**DOI:** 10.3390/cells9102281

**Published:** 2020-10-13

**Authors:** Katja Zappe, Margit Cichna-Markl

**Affiliations:** Department of Analytical Chemistry, Faculty of Chemistry, University of Vienna, 1090 Vienna, Austria; katja.zappe@univie.ac.at

**Keywords:** ABC transporter, DNA methylation, cancer, multidrug resistance, cancer therapy

## Abstract

ATP-binding cassette (ABC) transporters play a crucial role in multidrug resistance (MDR) of cancers. They function as efflux pumps, resulting in limited effectiveness or even failure of therapy. Increasing evidence suggests that ABC transporters are also involved in tumor initiation, progression, and metastasis. Tumors frequently show multiple genetic and epigenetic abnormalities, including changes in histone modification and DNA methylation. Alterations in the DNA methylation status of ABC transporters have been reported for a variety of cancer types. In this review, we outline the current knowledge of DNA methylation of ABC transporters in cancer. We give a brief introduction to structure, function, and gene regulation of ABC transporters that have already been investigated for their DNA methylation status in cancer. After giving an overview of the applied methodologies and the CpGs analyzed, we summarize and discuss the findings on aberrant DNA methylation of ABC transporters by cancer types. We conclude our review with the discussion of the potential to target aberrant DNA methylation of ABC transporters for cancer therapy.

## 1. Introduction

Resistance to anti-cancer drugs is a major obstacle in chemotherapy, resulting in limited effectiveness or even failure of therapy [1]. Drug resistance can be intrinsic or acquired. Intrinsic resistance exists before starting treatment, whereas acquired resistance arises during therapy [2]. Acquired resistance is particularly challenging, because tumors commonly become resistant not only to the administered drug, but to a broad spectrum of drugs differing in structure and mode of action [3]. Multidrug resistance (MDR) is multifactorial, involving mechanisms in the cancer cell and in the tumor microenvironment [4]. In particular, the interplay of tumor and tumor microenvironment is essential [5]. Factors at the cellular level include uptake, inactivation and efflux of drugs, the availability of molecular drug targets and DNA repair capacity [6]. Microenvironment-related factors are linked to the composition of the extracellular matrix, e.g., the abundance of cancer-associated fibroblasts, the recruitment of immune cells and the development of vascular networks [7].

Overexpression of ABCB1, ABCC1, and/or ABCG2 is a major cause of MDR in cancer. ABCB1, ABCC1, and ABCG2 are ATP-binding cassette (ABC) transporters functioning as efflux pumps, lowering intracellular accumulation of various anti-cancer drugs [8,9,10]. However, ABC transporters also transport a variety of endogenous substances across membranes, including phospholipids and cholesterol [11,12,13]. Since dysregulation of lipid homeostasis is considered an important factor in carcinogenesis [14,15,16], increasing evidence suggests that altered expression of ABC transporters not only contributes to MDR, but also to initiation, progression, and metastasis of cancer [11,12,13,17].

Carcinogenesis is a multistep process, driven by the accumulation of genetic and epigenetic alterations [18,19]. Changes in DNA methylation, the most widely studied epigenetic modification, occur more frequently than classical transforming events such as gene mutations and even may precede them [20]. Together with post-translational modifications (PTMs) of histone proteins, DNA methylation dynamically regulates chromatin accessibility and thus gene transcription [21,22].

Compared to normal cells, cancer cells frequently show global genomic hypomethylation, largely due to a loss of methylation of CpG dinucleotides (CpGs) in repetitive elements [23]. The extent of global genomic hypomethylation has been found to increase with tumor progression [24]. In contrast to repetitive elements, the promoter region of tumor suppressor genes is frequently hypermethylated in cancer cells [25]. Hypermethylation of the promoter region commonly leads to transcriptional inactivation of genes [26].

Aberrant DNA methylation of genes involved in cell cycle, DNA repair, toxic catabolism, cell adherence, apoptosis, and/or angiogenesis [23], but also of ABC transporters, has been detected in a variety of cancer types. Most studies investigating DNA methylation of ABC transporters in cancer are limited to *ABCB1*, *ABCC1*, and *ABCG2*, only few studies hint at aberrant DNA methylation of other members of the ABC transporter family.

In this review, we outline the current knowledge of DNA methylation of ABC transporters in cancer. We start by giving a brief introduction to structure, function, and gene regulation of ABC transporters. We only address ABC transporters that have already been investigated for their DNA methylation status in cancer. After giving an overview of the applied methodologies and the CpGs analyzed, we summarize and discuss the findings on aberrant DNA methylation of ABC transporters by cancer types and the potential to target aberrant DNA methylation of ABC transporters for cancer therapy.

## 2. Structure, Function, Expression, and Gene Regulation of ABC Transporters

ATP-binding cassette (ABC) transporters are ubiquitous and highly conserved membrane proteins that use the energy of ATP hydrolysis to transport exogenous and endogenous substances unidirectionally across membranes [27]. In eukaryotes, ABC transporters are exporters, with very few exceptions [28]. They commonly consist of two transmembrane domains (TMDs), embedded in the membrane bilayer, and two nucleotide binding domains (NBDs), located in the cytoplasm [29]. NBDs are involved in binding and hydrolysis of ATP, whereas TMDs determine ligand specificity [30]. Binding and hydrolysis of ATP lead to a switch between an outward- and inward-facing conformation of the TMDs, enabling the unidirectional transport across the lipid bilayer [28].

The number of human ABC transporters given in literature and databases ranges from 48 to 51, depending on whether pseudogenes are included or not. Based on sequence homology and structural organization, the ABC transporters have been grouped in seven subfamilies (ABCA–ABCG) [31,32].

### 2.1. ABCB1

ABCB1 (multidrug resistant protein 1, MDR1; P-glycoprotein, P-gp) is one of eleven members of the ABCB subfamily. It is a full transporter, consisting of two NBDs and two TMDs [33]. ABCB1 exports more than 200 substances of structural diversity, ranging from hydrophobic and amphipathic to cationic molecules [34,35,36]. Substrates include a variety of biologically active substances from plants, endogenous compounds like steroid hormones, but also drugs, e.g., anthracyclines (doxorubicin, daunorubicin), alkaloids (vincristine, vinblastine), and immunosuppressive agents (cyclosporine, tacrolimus) [35]. The broad substrate specificity results from the existence of multiple overlapping, transport-active binding sites [37,38].

ABCB1 is expressed in tissues having barrier function, ranging from epithelia of the liver, kidney, small and large intestine, to capillary endothelial cells in the brain, ovary, and testis [39]. In the placenta, it prevents toxins and drugs present in the maternal blood from entering fetal circulation, with ABCB1 expression decreasing with gestational age [40].

The human *ABCB1* gene is located on chromosome 7q21.12 and consists of 29 exons [41], with exon 1 and exon 2 being untranslated [42]. There are two promoters in the *ABCB1* gene. The promoter mainly located in exon 2 and intron 2 is commonly called “downstream” or “proximal” promoter, the promoter located upstream of exon 1 “upstream” or “distal” promoter. The downstream promoter has been identified to be the major promoter [43]. The upstream promoter is usually inactive in normal tissues. It has, however, been found to be active in tumors of patients suffering from acute lymphoblastic leukemia [44] or breast cancer [45]. The downstream promoter lacks a TATA box but contains an initiator (inr) element, two GC boxes, and an inverted CCAAT box. There are binding motifs for a variety of transcription factors, including Sp1, AP-1, C/EBPβ, and p53 [46].

Constitutive ABCB1 expression is predominantly regulated transcriptionally by binding of transcription factors [47]. Increasing evidence suggests that polymorphisms in the *ABCB1* gene may also affect ABCB1 expression and function [48,49]. The 3435T allele of the synonymous single nucleotide polymorphism (SNP) rs1045642 (3435C > T, exon 26) has been associated with significantly lower ABCB1 mRNA and protein levels in kidney and liver tissues, most likely due to decreased *ABCB1* mRNA stability [48,50]. rs1045642 is in strong linkage disequilibrium with the SNPs rs1128503 (1236C > T, exon 12) and rs2032582 (2677G > T/A, exon 21), with the haplotypes 1236C-2677G-3435C and 1236T-2677T-3435T being most abundant [51].

Upregulation of *ABCB1* has been reported for a variety of cancer types including lung, colon, kidney, adrenal gland, liver, pancreas, and hematological malignancies [52,53]. ABCB1 overexpression confers resistance to anthracyclines (e.g., doxorubicin and daunorubicin), epipodophyllotoxins (e.g., etoposide and teniposide), campothecins, *Vinca* alkaloids (e.g., vincristine and vinblastine), colchicine, and taxanes (e.g., paclitaxel and docetaxel) [54]. ABCB1 overexpression in MDR cancer cells is frequently associated with altered expression or activity of transcription factors, but also with gene rearrangements and mutations in the *ABCB1* promoter [47]. Recently, *ABCB1* has been found to be upregulated through fusion of *ABCB1* with *SLC25A40*, the gene upstream of *ABCB1*, in drug resistant high-grade serous ovarian and breast cancer [55]. Overexpression of ABCB1 is frequently linked to hypomethylation of the *ABCB1* promoter (see Section 4). There are also studies hinting at post-transcriptional regulation of ABCB1 expression by miRNAs [56].

### 2.2. ABCC1

ABCC1 (multidrug resistance-associated protein 1, MRP1) is a member of the ABCC subfamily, comprising 13 ABC transporters in total. ABCC1 consists of two NBDs and three TMDs [57]. It has an affinity for structurally different endogenous and exogenous substances, including organic anions, and their metabolites [58]. Its main endogenous substrates are leukotriene C4 and glutathione, the latter in both its reduced and oxidized form [59]. A variety of drugs is exported by ABCC1, e.g., *Vinca* alkaloids, anthracyclines, and etoposide [60]. ABCC1 is expressed in several tissues including liver, kidney, intestine, and brain [61].

The human *ABCC1* gene, located on chromosome 16p13.11, comprises 31 exons. The promoter lacks a TATA box but contains several overlapping GC boxes, showing binding motifs for Sp1 and Sp3 [57,62,63]. Constitutive ABCC1 expression is predominantly regulated transcriptionally by binding of transcription factors [47]. Numerous SNPs have been identified in the *ABCC1* gene, however, to date none of them has been associated with altered ABCC1 expression [64].

ABCC1 overexpression has been detected in various solid cancer types, including small cell lung carcinoma, prostate and breast cancer as well as childhood neuroblastoma, but also in hematological malignancies [65]. High ABCC1 levels have been linked to resistance to anthracyclines (e.g., doxorubicin), *Vinca* alkaloids (e.g., vincristine), epirubicin, etoposide, and methotrexate [54]. *ABCC1* gene amplification is considered to play a major role in MDR cell lines overexpressing ABCC1 [57].

### 2.3. ABCG2

ABCG2 (breast cancer resistance protein, BCRP; mitoxantrone resistance protein, MXR) belongs to the ABCG subfamily, together with four other ABC transporters. ABCG2 is a half transporter, consisting of only one NBD and one TMD. It has been found to form homodimers or even higher order oligomers to become fully functional [66,67].

ABCG2 is a very versatile transporter, exporting both hydrophobic and hydrophilic exogenous and endogenous substances as well as their phase II metabolites (sulfate and glucuronide conjugates) [68]. Analog to ABCB1, multiple drug binding sites have been identified for ABCG2 [67,69]. ABCG2 is highly expressed in almost all tissues with secretory or barrier function, e.g., placenta, central nervous system, small and large intestine, stomach, lung, kidney, prostate, and testis [70].

The human *ABCG2* gene is located on chromosome 4q22.1 and comprises 16 exons. The *ABCG2* promoter lacks a TATA box but contains a CAAT box and five binding sites for Sp1 [71]. In addition, an estrogen response element (ERE) has been identified in the promoter [72]. Transcription factor binding is the main mechanism regulating constitutive ABCG2 expression. The missense SNP rs2231142 (421 C > A, exon 5) has been associated with altered ABCG2 expression in in vitro and in vivo studies [73,74].

Elevated ABCG2 levels have been observed in various hematological malignancies (e.g., acute lymphoblastic leukemia (ALL) and chronic myeloid leukemia (CML)) and solid cancers (e.g., non-small cell lung cancer (NSCLC), ovarian and breast cancer) [75]. The spectrum of anti-cancer drugs ABCG2 confers resistance to overlaps with that of ABCB1. ABCG2 overexpressing cells are resistant to anthracyclines (e.g., doxorubicin and daunorubicin), epipodophyllotoxins (e.g., etoposide), mitoxantrone, irinotecan, and topotecan. In contrast to ABCB1, ABCG2 does not refer resistance to *Vinca* alkaloids and taxanes [54]. In some cancers, e.g., leukemia, ABCB1 and ABCG2 are commonly co-expressed. However, ABCB1 and ABCG2 have been found to function independently and additively in a cell line model expressing high levels of both ABC transporters [76].

*ABCG2* gene amplification plays an important role in ABCG2 overexpression in MDR cancer cells [77,78]. *ABCG2* gene amplification has been reported for a variety of cancer types, e.g., breast and colorectal cancer, and glioblastoma [36]. There is growing evidence that DNA methylation contributes to regulation of *ABCG2* (see Section 4). miRNAs (miR-132-3p, miR-212-3p) have been found to be involved in post-transcriptional regulation of *ABCG2* in clear cell renal cell carcinoma [79].

Increasing evidence suggests a crucial role of ABCG2 in intrinsic MDR of cancer stem cells [80,81], a small cell population in cancers, sharing several characteristics of normal stem cells. These characteristics include relative quiescence, the ability to self-renew and resistance to apoptosis [82,83]. Due to these properties, cancer stem cells are assumed to be responsible for recurrence, relapse, and metastasis of tumors [84,85].

### 2.4. ABCA1

ABCA1 (cholesterol efflux regulatory protein, CERP) belongs to the ABCA subfamily, consisting of 12 ABC transporters. Like other members of this subfamily, ABCA1 is a full transporter, consisting of two NBDs and two TMDs. It plays a crucial role in cellular cholesterol homeostasis by mediating the export of cholesterol to lipid-poor apolipoprotein, leading to the formation of high density lipoprotein (HDL) [86,87]. ABCA1 is expressed in macrophages, liver, small intestine, and brain [88].

The human *ABCA1* gene, located on chromosome 9q31.1, consists of 50 exons [89], with exon 1 and 2 encoding the 5′ untranslated region (UTR). The promoter lacks a CAT box, but contains a TATA box and several binding sites for transcription factors, including Sp1, NF-kB, AP-1, AP-2, and hepatocyte nuclear factor (HNF)-3β. In addition, there are three E box motifs and a cholesterol regulatory element [89].

ABCA1 expression is regulated at the transcriptional, post-transcriptional, and post-translational level. The nuclear receptors liver X receptor alpha (LXRa) and liver X receptor beta (LXRb) are involved in ABCA1 regulation at the transcriptional level [88]. miR-33a, miR-145, miR-148a, and miR-302a have been reported to regulate ABCA1 post-transcriptionally [90]. Several protein kinases regulate ABCA1 activity at the post-translational level, including protein kinase A, protein kinase C, Janus kinase 2, and casein kinase [88].

ABCA1 has been reported to be upregulated at the mRNA and protein level in triple negative breast cancer [91] and ovarian cancer [92], but downregulated in prostate cancer [93]. Higher ABCA1 levels have been associated with resistance to 5-fluorouracil, paclitaxel, etoposide, carboplatin, and mitoxantrone [54].

### 2.5. ABCB6

ABCB6 forms the ABCB subfamily, together with ABCB1 and nine other ABC transporters. ABCB6 is a half transporter that functions as a homodimer. However, compared to other half transporters, it contains one additional TMD [94].

ABCB6 is involved in porphyrin transport and iron homeostasis [94]. It is widely expressed in the heart, liver, skeletal muscles, red blood cells, and the skin [95].

The human *ABCB6* gene is located on chromosome 2q35 and comprises 19 exons. The *ABCB6* promoter is rich in CpGs, contains a TATA box and has multiple binding motifs for Sp1 and p53. In addition, there are six glucocorticoid (GR) response elements [96]. The mechanisms involved in ABCB6 regulation remain to be elucidated [94].

Overexpression of ABCB6 has been observed in hepatocellular cancer [97]. ABCB6 upregulation has been associated with resistance to 5-fluorouracil, doxorubicin, paclitaxel, irinotecan, and oxaliplatin [54].

### 2.6. ABCC6

ABCC6 (multidrug resistance-associated protein 6, MRP6) is a member of the ABCC subfamily. Analog to ABCC1, ABCC6 consists of two NBDs and three TMDs [98]. In *ABCC6*-transfected Chinese hamster ovary cells, ABCC6 has been found to transport glutathione *S* conjugates but not glucuronide conjugates [99]. The *ABCC6*-transfected cells showed low resistance to etoposide, teniposide, doxorubicin, and daunorubicin [99]. ABCC6 is known to be involved in the regulation of tissue calcification in mammals [100]. It is primarily expressed in the liver and the kidneys, lower levels have been found in lung, intestines, retina, skin, and vessel walls [101].

The human *ABCC6* gene is located on chromosome 16p13.11 and consists of 31 exons. It shows 44% sequence identity with the human *ABCC1* gene [102]. The promoter contains binding motifs for several transcription factors, including AP-2, USF-1, NF-kB, and epidermal growth receptor [103].

ABCC6 is, at least in part, regulated transcriptionally by transcription factor binding [103,104].

### 2.7. ABCD1

ABCD1 (adrenoleukodystrophy protein, ALDP) belongs to the small ABCD subfamily, comprising only four ABC transporters. All members of this subfamily are half transporters, consisting of only one NBD and one TMD [105]. ABCD1 functions as homodimer, but a heterodimeric structure has also been suggested [105].

ABCD1 transports long and very long chain fatty acids and their coenzyme A esters into peroxisomes [105]. High ABCD1 expression levels have been reported for the adrenal gland, heart, intestine, kidney, liver, lung, placenta, and testis [106].

The human *ABCD1* gene is found on chromosome Xq28 and contains ten exons. Mutations in *ABCD1* cause X-linked adrenoleukodystrophy, a progressive neurodegenerative disease, characterized by the accumulation of very long chain fatty acids in plasma and tissues [107].

Altered ABCD1 expression has been reported for melanoma, breast, and renal cell carcinoma [108].

### 2.8. ABCG5

ABCG5 is a half transporter belonging to the ABCG subfamily. It has to form a heterodimer with ABCG8, a member of the same subfamily, to be functional [109,110]. The ABCG5/ABCG8 heterodimer, sterolin, plays a role in the removal of excess cholesterol into the bile [109,111]. Both ABCG5 and ABCG8 are predominantly located on the apical membranes of enterocytes and hepatocytes [109].

The human *ABCG5* and *ABCG8* genes are located in close proximity, oppositely orientated, on chromosome 2p21. Their head-to head orientation suggests that the two genes share a bidirectional promoter [112]. Each gene comprises 13 exons. *ABCG5* and *ABCG8* are regulated at the transcriptional level by the nuclear receptor liver receptor homolog-1 (LRH-1) [113].

Mutations in *ABCG5* and *ABCG8* cause sitosterolemia, a rare autosomal recessive disorder, characterized by hyperabsorption of plant sterols such as sitosterol [114].

## 3. DNA Methylation Analysis of ABC Transporters in Cancer

Studies investigating DNA methylation of ABC transporters in cancer have been performed on cancer cell lines, MDR cell line models, and/or clinical samples. A variety of methodologies have been applied for DNA methylation analysis, including restriction enzyme-based methods, methylation-specific polymerase chain reaction (MSP), methylation-sensitive high resolution melt (MS-HRM) analysis, pyrosequencing (PSQ), bisulfite sequencing (BS), and microarray-based methods.

Restriction enzyme-based methods frequently involve the restriction endonuclease pair *Hpa*II/*Msp*I. Both cleave the same recognition sequence (5′-CCGG-3′), but *Hpa*II in a methylation-sensitive (only if the internal cytosine is unmethylated), *Msp*I in a methylation-insensitive mode. The profiles obtained after polymerase chain reaction (PCR) amplification and separation by agarose gel electrophoresis show whether the CpG within the recognition sequence was methylated or unmethylated [115]. The principle of combined bisulfite restriction analysis (COBRA) is similar, but the DNA is treated with sodium bisulfite prior to PCR amplification [116]. In this pre-treatment step, unmethylated cytosine is converted to uracil (which is then replaced by thymine during PCR), whereas methylated cytosine remains unchanged [117]. MSP, PSQ, MS-HRM, BS, and microarray-based methods also require pre-treatment of DNA with sodium bisulfite. MSP makes use of methylation-specific primers for amplification of the target region. The PCR products are either detected on an agarose gel, or in real-time by using a fluorescent dye [118]. MS-HRM is also based on amplification of the target region by PCR, but in contrast to MSP, methylation-insensitive primers are used and the PCR products are subjected to a high resolution melting step. The methylation status can be assessed from the melting profiles of the PCR products, by comparison with melting profiles of PCR products obtained for calibration standards (methylated and unmethylated DNA, and mixtures thereof) [119].

MSP and MS-HRM only provide information on the average methylation status across the CpGs in the target region. This limitation can be overcome by applying PSQ, in which the primary structure of a single-stranded DNA fragment is determined by synthesis of the complementary strand [120]. BS yields information on DNA methylation patterns in single molecules, either by subcloning followed by Sanger sequencing, or by applying next generation sequencing (NGS) technologies [121]. Methylation Bead Chips allow genome-wide methylation analysis of 450,000 or even more than 850,000 CpGs at single-nucleotide resolution by using pairs of hybridization probes, one being complementary to the converted, methylated and the other one to the converted, unmethylated sequence [122].

**Figure 1:**
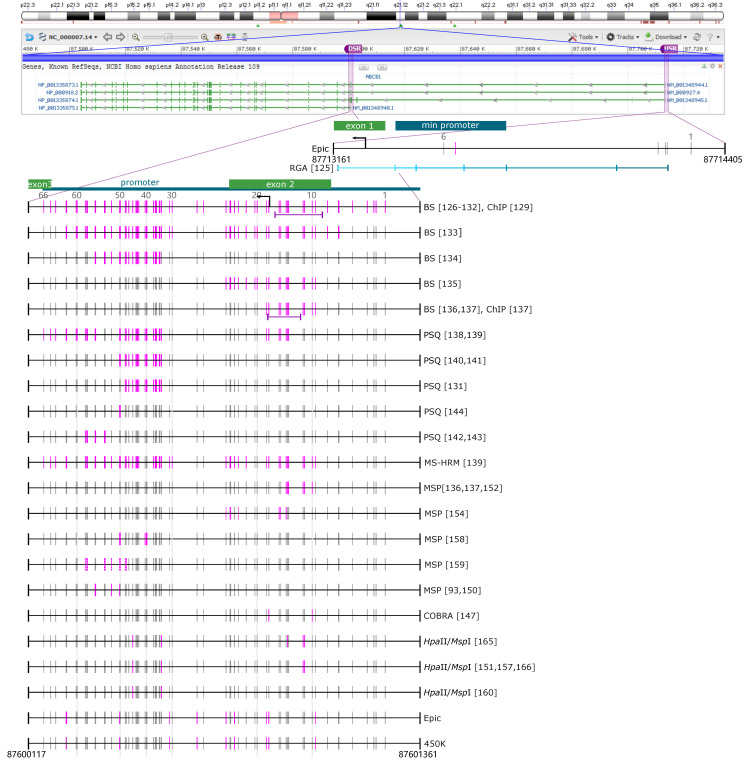
Schematic representation of the human *ABCB1* gene (chromosome 7q21.12, reverse strand). Transcripts are shown as merged transcript and coding sequence (CDS) pairs. The distal upstream region (USR, GenBank NC_000007.14: range 87,713,161 to 87,714,405) contains six CpGs, the minimal promoter [125] (identified by reporter gene assay (RGA), blue horizontal bars), exon 1 (GenBank NG_011513.1), and the alternative transcription start site ([125], GenBank) marked by an arrow. The proximal downstream region (DSR, range 87,600,117 to 87,601,361) contains 66 CpGs, exons 2 and 3 (GenBank), and the major transcription start site ([126], GenBank) marked by an arrow. The promoter ranges from 87,600,162 to 87,601,361 [126]. CpGs investigated are highlighted in pink. CpGs that have only been analyzed for their unmethylated or methylated status by MSP are represented by dotted or dashed lines, respectively. Purple horizontal bars mark regions investigated by chromatin immunoprecipitation (ChIP). Methylation analysis has been performed by bisulfite sequencing (BS), pyrosequencing (PSQ), Infinium Methylation BeadChips (450K, Epic), methylation-sensitive high resolution melting (MS-HRM), methylation-specific PCR (MSP), combined bisulfite restriction analysis (COBRA) or *Hpa*II*/Msp*I restriction prior to PCR.

**Figure 2:**
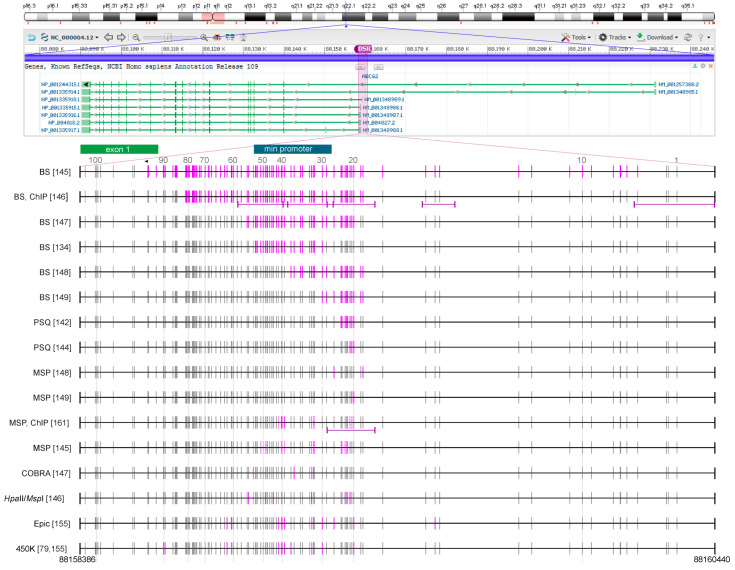
Schematic representation of the human *ABCG2* gene (chromosome 4q22.1, reverse strand). Transcripts are shown as merged transcript and coding sequence (CDS) pairs. The downstream region (DSR, GenBank GRCh38.p13 NC_000004.12: range 88,158,386 to 88,160,440) contains 102 CpGs, the minimal promoter [71], exon 1 (GenBank NG_032067.2), and the transcription start site (GenBank) marked by an arrow. CpGs investigated are highlighted in pink. CpGs that have only been analyzed for their unmethylated or methylated status by MSP are represented by dotted or dashed lines, respectively. Purple horizontal bars mark regions investigated by chromatin immunoprecipitation (ChIP). Methylation analysis has been performed by bisulfite sequencing (BS), pyrosequencing (PSQ), Infinium Methylation BeadChips (450K, Epic), methylation-specific PCR (MSP), combined bisulfite restriction analysis (COBRA) or *Hpa*II/*Msp*I restriction prior to PCR.

There is increasing evidence that promoter regions may be methylated heterogeneously [123] and that specific CpGs may be more biologically and/or clinically relevant than others [124]. For comparability reasons, we tried to figure out number and position of the CpGs targeted in the individual studies on *ABCB1* (Figure 1) and *ABCG2* (Figure 2), the ABC transporters most frequently investigated with respect to DNA methylation in cancer. However, re-tracing of target CpGs was a time-consuming and challenging task, because information on the target region was not always as clear as hoped for. In some cases, the primer sequences given turned out to be incorrect or even not specified. Studies that did not allow the identification of the target CpGs were excluded from this review.

Thirty-one studies have investigated the downstream region (DSR) of *ABCB1* (Figure 1). Among them, seven studies have targeted each of the 66 CpGs in the *ABCB1* downstream promoter by BS [126,127,128,129,130,131,132]. Other studies applying BS have targeted 56 [133], 25 [134], 19 [135], or eleven [136,137] CpGs. The PSQ assays applied allowed the determination of the methylation status of 39 [138,139], 20 [140,141], 18 [131], seven [142,143], or two [144] CpGs. However, not all of these studies have provided methylation data for each single CpG, which hampers comparability of results obtained in different studies (see Section 4). Most commonly, CpGs 32, 46, and 51 of the *ABCB1* promoter have been targeted. CpGs 9−19 and 32−59 have been analyzed in ≥ 12 studies (Figure 1).

Eleven studies report *ABCG2* methylation data (Figure 2). Analog to *ABCB1*, higher numbers of CpGs (91 [145], 66 [146], 38 [147], 25 [134], 21 [148], or 13 [149]) have been analyzed by BS than by other methodologies. CpGs 20−42 have been analyzed in ≥ 5 studies (Figure 2).

## 4. Aberrant DNA Methylation of ABC Transporters in Cancer

Aberrant DNA methylation has already been detected in various solid and hematological cancers. In the following, we summarize and discuss these findings, starting with thoracic malignancies [132,142,145,147,150], continuing with breast cancer [129,130,131,138,139,140,141,142,147,151,152], colorectal cancer [127,143,148], upper gastrointestinal cancer [133,134,153,154,155,156], genitourinary cancers [79,93,136,137,146,157,158,159,160,161,162,163], gynecological cancers [139,142,147,164], tumors of the central nervous system [144], ending with hematological malignancies [126,127,128,133,135,142,147,149,165,166]. Table 1, Table 2 and Table 3 summarize findings of studies on cancer cell lines, MDR cell line models, and clinical samples, respectively.

Since DNA methylation analysis of *ABCB1* has been focused on the downstream promoter (Section 3), discussion on DNA methylation of *ABCB1* refers to the downstream promoter, unless otherwise indicated.

### 4.1. Lung Cancer

Nakano et al. have investigated the methylation status of the *ABCG2* promoter in non-small cell lung cancer (NSCLC) (NCI-H358, NCI-H441, NCI-H460) and small cell lung cancer (SCLC) (NCI-H69, PC-6) cell lines [145]. NCI-H69 and PC-6 cells did not express ABCG2, whereas NCI-H358, NCI-H441, and NCI-H460 cells showed moderate ABCG2 expression. ABCG2 expression has been found to be inversely correlated with *ABCG2* promoter methylation status in both lung cancer subtypes. The involvement of promoter methylation in *ABCG2* gene regulation has been confirmed by treatment of PC-6 cells with the DNA methyltransferase inhibitor 5-aza-2′-deoxycytidine (5-aza-dC). 5-aza-dC induced ABCG2 expression at the mRNA and protein level in a dose-dependent manner [145].

Spitzwieser et al. have determined promoter methylation levels of *ABCB1*, *ABCC1*, and *ABCG2* in seven cancer cell lines, including five NSCLC (A549, HCC827, NCI-H520, NCI-H1703, SW 1573) and two SCLC (DMS 114, GLC-4) cell lines [142]. The *ABCC1* promoter was unmethylated in each of the cell lines. The *ABCB1* promoter has been found to be unmethylated (DMS 114), lowly methylated (<25%; A549, NCI-H520, GLC-4) or highly methylated (>75%; HCC827, NCI-H1703, SW 1573). The *ABCG2* promoter was lowly (<25%) methylated in four (HCC827, NCI-H520, DMS 114, GLC-4), moderately methylated in two (A549, SW 1573), and highly methylated (>75%) in one (NCI-H1703) cell line. These results indicate that in the lung cancer cell lines investigated, promoter methylation of *ABCB1*, *ABCC1*, and *ABCG2* was not associated with the lung cancer subtype.

The role of ABC transporter methylation in acquisition of an MDR phenotype has been investigated in various MDR models of both NSCLC [132,142,147] and SCLC cell lines [142,145]. In A549/DDP, an MDR subline of A549 resistant to the cisplatin analog diaminedichloroplatinum, overexpression of ABCB1 at both the mRNA and protein level was associated with hypermethylation of the *ABCB1* promoter [132]. In another MDR cell line model of A549, A549/K1.5, established by selecting A549 cells against triazoloacridone C-1305, overexpression of ABCG2 has been found to be caused by gene amplification, without any changes in *ABCG2* promoter methylation [147]. In SW 1573/2R160, a doxorubicin-resistant subline of SW 1573 overexpressing ABCB1 and ABCC1, the *ABCB1* promoter was significantly lower methylated compared to the parental cell line [142]. In addition, amplification of both the *ABCB1* and the *ABCC1* gene has been observed in SW 1573/2R160 cells [142]. In the same study, ABCC1 overexpression in GLC-4/adr, a doxorubicin-resistant subline of GLC-4, was caused by *ABCC1* gene amplification. DNA methylation changes have not been observed for the *ABCC1* promoter, but for some CpGs in the *ABCB1* and *ABCG2* promoters [142]. Overexpression of ABCG2 in the MDR cell line model PC-6/SN2-5H, established by continuous exposure of PC-6 cells to SN-38, the active metabolite of the DNA topoisomerase I inhibitor irinotecan, was associated with hypomethylation of the *ABCG2* promoter [145].

Findings for MDR cell line models suggest that changes in *ABCB1* and *ABCG2* promoter methylation are involved in acquiring MDR in lung cancer. Whether DNA methylation changes occur in *ABCB1* or *ABCG2* and whether the respective promoter is hyper- or hypomethyated, seems to depend on the cell line and the substance it was selected for.

Gao et al. have determined the *ABCB1* promoter methylation status in tumor samples from 36 patients with bronchioloalveolar carcinoma, a subtype of lung adenocarcinoma, and ten normal lung tissue samples from patients with inflammatory pseudotumors [150]. The *ABCB1* promoter has been found to be methylated in 26 of the 36 tumors, but also in each of the control samples. In tumor tissues, the methylation status of the *ABCB1* promoter was inversely correlated with ABCB1 expression. *ABCB1* promoter methylation was not associated with smoking habit, lymph node metastasis, tumor size, tumor stage, recurrence rate, or survival rate. Findings of this study suggest that *ABCB1* promoter methylation cannot be considered a potential diagnostic, predictive or prognostic biomarker in lung cancer [150]. In lung adenocarcinoma from 20 patients, higher ABCB1 mRNA and protein levels have been found than in normal adjacent lung tissues [132]. Upregulation of ABCB1 was associated with hypermethylation of the *ABCB1* promoter.

In summary, three studies have investigated *ABCB1* and three studies *ABCG2* promoter methylation in lung cancer. These studies hint at aberrant DNA methylation of *ABCB1* and *ABCG2* promoters in lung cancer. Two studies have found *ABCB1* promoter methylation levels to be correlated with ABCB1 expression levels [132,150]. However, results from different studies are not in accordance. One has reported a direct [132] and the other one an inverse [150] correlation between promoter methylation and gene expression. Thus, the role of *ABCB1* and *ABCG2* methylation in lung cancer remains unclear to date.

### 4.2. Breast Cancer

By DNA methylation analysis of the *ABCB1*, *ABCC1*, and *ABCG2* promoters in three breast cancer cell lines (MCF7, luminal A subtype; ZR-75-1, luminal B subtype; MDA-MB-231, triple negative subtype), the *ABCC1* promoter has been found to be unmethylated in each of the cell lines [142]. In all cell lines, the *ABCB1* promoter was higher methylated than the *ABCG2* promoter [142].

Several studies have investigated *ABCB1* promoter methylation in MDR MCF7 sublines to obtain data on the mechanism of acquiring an MDR phenotype [129,130,131,151]. Comparison of the DNA methylation status of the *ABCB1* promoter in MCF7 cells, lacking ABCB1 expression, and doxorubicin-resistant sublines, overexpressing ABCB1, has shown substantially lower *ABCB1* promoter methylation in the sublines compared to the parental cell line [129,151]. In addition to differences in the extent of DNA methylation, differences in chromatin structure have been observed. In the MDR cell model MCF7/ADR, the histone tails have been found to be acetylated, resulting in a transcriptionally active chromatin, whereas in the parental cell line, there was a lack of acetyl marks, indicating that the chromatin was repressed [129]. The set of MDR MCF7 sublines used by Reed et al. [130,131] consisted of MCF7/TXT [130], MCF7/DOX-2, MCF7/EPI, and MCF7/TAX-2 [131], established by selecting MCF7 cells with increasing concentrations of docetaxel, doxorubicin, epirubicin, or paclitaxel, respectively. With the exception of MCF7/DOX-2, acquisition of the MDR phenotype was associated with upregulation of ABCB1. In MCF7/TXT cells, the drug dose correlated with the extent of hypermethylation of the *ABCB1* downstream promoter, amplification of the *ABCB1* gene and ABCB1 expression levels [130]. However, in case of MCF7/EPI and MCF7/TAX-2 cells, acquisition of MDR was associated with hypomethylation of the *ABCB1* promoter, in the absence of gene amplification [131]. Interestingly, at the highest selection dose, the upstream promoter was used instead of the downstream promoter [130,131]. Results obtained by BS suggested allele-specific methylation of the *ABCB1* downstream promoter and allele-specific regulation of *ABCB1* promoter usage in drug-resistant MCF7 cells. However, the lack of both ABCB1 upregulation and *ABCB1* promoter hypomethylation in MCF7/DOX-2 [131] contradicts to results obtained for other doxorubicin-resistant sublines of MCF7 [129,151]. The discrepancy in DNA methylation data between the studies by David et al. [129] and Reed et al. [131] cannot be explained by methodological differences, since in both studies the identical 66 CpGs (Figure 1) have been targeted by the same BS assay. In two other sublines of MCF7, MCF/MR and MCF7-FLV1000, established by selecting MCF7 cells against mitoxantrone and flavopiridol, respectively, Bram et al. have found ABCG2 overexpression to be associated with *ABCG2* gene amplification, but not with any changes in promoter methylation [147].

Six studies have determined DNA methylation levels of *ABCB1* in clinical samples from breast cancer patients [138,139,140,141,142,152]. Notably, the *ABCB1* promoter has been found to be hypomethylated [152], hypermethylated [138,140,141,142] or equally methylated [139] in tumor tissues compared to normal breast tissues serving as controls. This inconsistency can, at least in part, be due to differences in DNA methylation analysis. In the study by Sharma et al. [152], reporting *ABCB1* promoter hypomethylation, the average methylation status across six CpGs has been determined by MSP. In the other studies [138,139,140,141,142], the methylation status of individual CpGs, most of them being downstream of the CpGs targeted in [152], has been determined by PSQ.

The study by Sharma et al. on tumor and serum samples from 100 patients with invasive ductal breast carcinoma (IDC) has investigated the potential of the *ABCB1* promoter methylation status as predictive or prognostic marker in breast cancer [152]. The *ABCB1* promoter has been found to be hypomethylated in 47% of the tumors and 44% of paired sera of IDC patients, but also in two of 15 paired normal breast tissues and three of 30 sera from healthy women. Hypomethylation of the *ABCB1* promoter in tumor and serum samples was associated with a shorter median overall survival of the patients [152].

With the aim to identify a DNA methylation based biomarker to stratify breast cancer patients for neoadjuvant treatment with doxorubicin, the methylation status in the promoter regions of 14 genes including *ABCB1* has been determined in 75 samples from locally advanced breast cancer [138]. Six normal breast tissues have been used as controls. In breast cancer tissues, aberrant promoter methylation has been observed for nine out of the 14 genes, including *ABCB1.* The *ABCB1* promoter has been found to be hypermethylated compared to normal breast tissues. The *ABCB1* promoter methylation status correlated with response to doxorubicin treatment, suggesting that it could be used to predict the response to doxorubicin. However, *ABCB1* promoter methylation was not correlated with ABCB1 expression [138].

By determining the methylation status of the promoter region of eleven genes including *ABCB1* in 27 ductal carcinoma in situ (DCIS), 28 small IDCs, 34 IDCs with a DCIS component, and 28 normal breast tissue samples, *ABCB1* promoter methylation was as frequently detected in DCIS, a non-invasive lesion of the breast, as in early invasive breast cancer [141]. Estrogen receptor (ER) positive tumors showed higher *ABCB1* methylation levels than ER negative tumors. In addition, *ABCB1* methylation was lower in highly proliferative tumors, suggesting a role for *ABCB1* methylation in breast cancer progression and outcome [141]. In a follow-up study, the DNA methylation status of 12 candidate genes including *ABCB1* has been determined in 238 breast cancer tissue samples from early premalignant DCIS to advanced metastatic breast cancer [140]. A difference in the methylation level between DCIS and invasive stage II tumors has been observed for six genes, including *ABCB1*. *ABCB1* was significantly higher methylated in late stage compared to early stage breast carcinoma [140]. 

Promoter methylation of *ABCB1*, *ABCC1*, and *ABCG2* has been investigated in tumor, tumor-adjacent, and tumor-distant tissues from 16 breast cancer patients and normal breast tissues from four healthy women [142]. The *ABCC1* promoter has been found to be unmethylated in all tissue samples. The *ABCB1* promoter was significantly higher methylated in tumors than in tumor-adjacent and tumor-distant tissues from the same patients and normal breast tissues of the control group, suggesting a role of *ABCB1* promoter methylation in breast carcinogenesis [142].

Vaclavikova et al. have determined the methylation status of the *ABCB1* promoter in tumor tissues from 83 breast carcinoma patients prior to chemotherapy and 112 patients after chemotherapy (34 treated with 5-fluorouracil and mitomycin, 78 with doxorubicin) [139]. In the samples collected prior to chemotherapy, but not in those collected after chemotherapy, methylation levels of the *ABCB1* promoter were inversely correlated with *ABCB1* mRNA levels. No difference in *ABCB1* methylation has been found between pre-treatment and post-treatment breast carcinoma samples, and between tumor samples and paired adjacent tissue samples serving as controls.

In summary, only two studies have investigated *ABCG2* promoter methylation in breast cancer, with none of them reporting aberrant methylation compared to normal breast cells/tissues. However, four out of six studies have found *ABCB1* promoter hypermethylation in breast cancer. In addition, hypomethylation of the *ABCB1* promoter in the MDR phenotype of breast cancer has been reported in four out of six studies. These findings suggest a role of *ABCB1* promoter methylation in breast carcinogenesis and in establishing the MDR phenotype.

### 4.3. Colorectal Cancer

To date, studies on DNA methylation of ABC transporters are limited to colorectal cancer cell (CRC) lines [127,148] and one MDR cell line model [143]. Baker et al. have found out that the chemotherapeutic drugs daunorubicin and etoposide could upregulate ABCB1 expression only in CRC cells in which the *ABCB1* promoter was almost unmethylated [127]. In the CRC cell line SW620, in which the *ABCB1* gene was already transcriptionally active, drug-induced upregulation of *ABCB1* was not associated with further changes in promoter methylation, but with increased histone acetylation [127]. In a study by Moon et al., 32 CRC cell lines have been investigated for their *ABCG2* expression and *ABCG2* promoter methylation levels [148]. DNA methylation analyses have been performed by MSP, targeting two CpGs (CpG 18 and 28), and by BS, yielding information on 21 CpGs (CpG 18–38) (Figure 2). In general, higher *ABCG2* promoter methylation levels have been obtained by MSP than by BS (Table 1). These data clearly indicate that the methodology has an impact on the DNA methylation levels determined. In three cell lines showing low *ABCG2* expression, SNU-C4, LS 174T, and NCI-H716, *ABCG2* expression was inversely correlated with *ABCG2* promoter methylation. Treatment of these cell lines with 5-aza-dC resulted in *ABCG2* promoter demethylation, upregulation of *ABCG2* and decreased anti-cancer drug sensitivity, indicating that in these cell lines, *ABCG2* promoter methylation played a role in *ABCG2* gene regulation.

To elucidate the underlying mechanism of limited effectivity of treating solid cancers with the thiosemicarbazone triapine, a triapine-resistant cell line, SW480/tria, has been established by selecting SW480 cells continuously with triapine [143]. SW480/tria cells have been found to overexpress *ABCB1*, and *ABCB1* overexpression was associated with *ABCB1* promoter hypomethylation.

### 4.4. Esophageal Cancer

Two recent studies have addressed DNA methylation of ABC transporters in esophageal squamous cancer, the predominant subtype of esophageal cancer, accounting for 80% of all patients [133,153]. Sumarpo et al. have been interested in the mechanism of ABCB1 upregulation in two taxane-resistant esophageal squamous cancer cell lines, RTE-1D, a docetaxel-resistant subline, and RTE-1P, a paclitaxel resistant cell line of TE-1 [133]. The DNA methylation status has been determined in two regions of the *ABCB1* promoter, with region 1 including exon 2A and 2B with the transcription start site from exon 2B, and region 2 being part of intron 2. Upregulation of ABCB1 has been found to be associated with hypomethylation of region 1, whereas the DNA methylation status of region 2 remained unchanged. However, hypomethylation of region 1 of the *ABCB1* promoter was not the only mechanism underlying ABCB1 upregulation. A gain in *ABCB1* gene copy number and changes in histone modification have also been detected [133].

In order to identify DNA methylation-driven genes in esophageal squamous cell cancer, Lu et al. have analyzed DNA methylation and transcriptome profiling data for 96 esophageal squamous cell carcinoma samples and three normal samples, downloaded from The Cancer Genome Atlas (TCGA) [153]. Twenty-six genes including *ABCD1* were aberrantly methylated and their methylation status was correlated with gene expression. Moreover, methylation and gene expression levels of *ABCD1* and two other genes have been found to be correlated with patients’ survival [153].

### 4.5. Gastric Cancer

To date, studies on DNA methylation changes of ABC transporters in gastric cancer are limited in number. Tahara et al. have determined the DNA methylation status of the *ABCB1* promoter in tumor tissues and paired non-neoplastic mucosa from 83 patients with gastric cancer [154]. In tumor tissues, the *ABCB1* promoter was significantly higher methylated than in non-neoplastic mucosa. Particularly high *ABCB1* promoter methylation levels have been found in intestinal cancers, in more advanced cancers, and in lymph vessel invasion-positive cancers [154].

Very recently, Ohmura et al. have investigated whether DNA methylation changes are linked to better prognosis and higher chemotherapy sensitivity of Epstein–Barr virus-associated gastric cancer (EBVGC), compared to other molecular subtypes [155]. By determining DNA methylation levels in gastric cells originating from an advanced EBVGC patient sensitive to chemotherapy and comparing the results with TCGA data, they have identified genes that had already been associated with cisplatin resistance and were differentially methylated in EBVGC cells compared to normal gastric cells. One of these genes was *ABCG2*, with the *ABCG2* promoter being hypermethylated in EBVGC cells. This finding hints at a contribution of *ABCG2* promoter hypermethylation to higher sensitivity to chemotherapy in EBVGC [155].

### 4.6. Hepatic Cancer

In a study by Tsunedomi et al. including tumor samples from 81 hepatocellular carcinoma patients, higher *ABCB6* mRNA levels have been found in hepatitis C virus (HCV)-related hepatic cancers with early intrahepatic recurrence compared to HCV-related cancers without early recurrence and the corresponding non-cancerous livers [156]. *ABCB6* mRNA levels inversely correlated with the methylation status of a CpG island in the promoter of *ABCB6.* This inverse correlation has also been observed in the hepatoma cell lines Hep 3B, Hep G2, HLE, HuH-6, HuH-7, and SK-HEP-1. These findings suggest that *ABCB6* methylation is a potential predictive biomarker for early intrahepatic recurrence of HCV-related hepatic cancers [156].

### 4.7. Pancreatic Cancer

Chen et al. have determined *ABCB1*, *ABCC1*, and *ABCG2* promoter methylation and expression levels in the pancreatic cancer cell line SW1990, its gemcitabine-resistant subline SW1990/GZ, and one normal pancreatic tissue serving as control [134]. *ABCB1*, *ABCC1*, and *ABCG2* mRNA levels were significantly higher in SW1990/GZ than in the parental cell line. However, the *ABCB1*, *ABCC1*, and *ABCG2* promoters have been found to be unmethylated in the gemcitabine-resistant subline and its parental cell line as well as in the normal pancreatic tissue, indicating that promoter methylation did not play a role in gene regulation of *ABCB1*, *ABCC1*, and *ABCG2*.

### 4.8. Renal Cancer

In a study by To et al. including three human sporadic clear cell renal carcinoma cell lines, CpGs 1–52 in the *ABCG2* promoter were highly methylated in the cell lines UOK121 and UOK143, resulting in transcriptional silencing. In the ABCG2 expressing cell line UOK181, the *ABCG2* promoter was unmethylated [146]. Hypermethylation of the *ABCG2* promoter in the cell lines UOK121 and UOK143 was associated with the methyl CpG binding domain proteins (MBDs) MBD2 and MeCP2, as well as with histone deacetylation and methylation of lysine at position 9 on histone H3 (H3K9). By treating UOK121 and UOK143 cells with 5-aza-dC, ABCG2 expression has been induced. These results suggest that both DNA methylation and histone modification play a role in ABCG2 regulation in renal cancer.

Reustle et al. aimed to elucidate the biological relevance and the regulatory mechanism of ABCG2 in clear cell renal cell carcinoma [79]. The study comprised five renal cancer cell lines (ACHN, A-498, Caki-1, Caki-2, and 786-O), a patient cohort from TCGA (453 patients), an own cohort (64 patients), and an independent set of transcriptome data (53 patients with metastases, who had been treated first-line with sunitinib, a multityrosine kinase inhibitor). *ABCG2* promoter methylation data have been shown for the five renal cancer cell lines, for 274 patients from the TCGA cohort, and for 16 patients from the own cohort. The cancer cell lines have been found to differ substantially in *ABCG2* promoter methylation. In contrast, cancer tissues from both the TCGA cohort and the own cohort showed similar DNA methylation levels, independent of their *ABCG2* mRNA levels. Aberrant *ABCG2* expression has been found to be regulated post-transcriptionally. ABCG2 mRNA and protein expression levels were inversely associated with cancer severity and patients’ survival.

### 4.9. Bladder Cancer

Tada et al. have analyzed 51 tumor tissue samples from bladder cancer patients to elucidate whether *ABCB1* gene expression and *ABCB1* promoter methylation levels were changed during chemotherapeutic treatment [157]. *ABCB1* mRNA levels were 3.5–5.7-fold higher in bladder cancers after chemotherapeutic treatment than those in untreated primary tumors. *ABCB1* mRNA levels have been found to be inversely correlated with the *ABCB1* promoter methylation status, indicating that *ABCB1* promoter methylation was involved in gene regulation.

A study by Yu et al. aimed at identifying a set of DNA methylation markers in urine sediments for the sensitive and specific detection of bladder cancer [158]. The methylation status of 59 tumor-associated genes has been determined in three bladder cancer cell lines, a cohort of cancer biopsies, and urine sediments. Twenty-one candidate genes have then been profiled in urine sediments from 132 bladder cancer patients, 23 age-matched patients with noncancerous urinary lesions, six neurologic diseases, and seven healthy volunteers. Cancer-specific hypermethylation in urine sediments has been reported for 15 genes, including *ABCC6*. Results suggest that the methylation profile of a set of eleven genes including *ABCC6* in urine sediment should allow the detection of bladder cancer with a sensitivity of 91.7% and a specificity of 87.6%. In addition, the set included the gene encoding fibrosis transmembrane conductance regulator (CFTR, ABCC7), another member of the ABC transporter subfamily C. However, ABCC7 is an untypical ABC transporter, functioning as an ATP-gated chloride channel [167].

### 4.10. Prostate Cancer

Studies on DNA methylation of ABC transporters in prostate cancer have been performed on prostate cancer cell lines and/or clinical samples [93,136,137,159,160,161,162,163]. Yegnasubramanian et al. have investigated the promoter methylation status of 16 cancer-related genes in two normal prostate and seven prostate cancer cell lines [159]. Five genes including *ABCB1* were hypermethylated in prostate cancer cell lines. In the same study, 25 benign prostate tissues, 73 primary prostate cancer tissues, and 91 metastatic prostate cancer tissues have been analyzed. The *ABCB1* promoter has been found to be hypermethylated in a large percentage of primary prostate cancers, whereas it was almost never methylated in benign tissues. Interestingly, samples of different stages of prostate cancer showed similar methylation levels [159]. Even in metastases, the methylation levels were similar to those of primary prostate cancers.

By analyzing four prostate cancer cell lines, Henrique et al. have found *ABCB1* promoter methylation levels to be inversely correlated with *ABCB1* mRNA levels [137]. However, exposure of the prostate cancer cell lines to 5-aza-dC and the histone deacetylases inhibitor trichostatin A indicated that downregulation of *ABCB1* was mainly caused by histone modifications, whereas promoter hypermethylation seemed to play a minor role. By determining *ABCB1* promoter methylation and expression in 121 prostate cancers, 37 high-grade prostatic intraepithelial neoplasia, 26 benign prostatic hyperplasia, and ten morphologically normal prostate tissue samples, frequency and levels of *ABCB1* promoter methylation increased from normal prostate tissue samples to high-grade prostatic intraepithelial neoplasia to prostate cancers [137].

In a study by Enokida et al., including 177 prostate cancer samples and 69 benign prostate hypertrophy samples, the *ABCB1* promoter has been found to be significantly more often methylated in prostate cancer samples than in benign prostate hypertrophy samples [136]. *ABCB1* promoter methylation was significantly associated with disease progression.

A study by Demidenko et al. has reported downregulation of eight (ABCA8, ABCB1, ABCC6, ABCC9, ABCC10, ABCD2, ABCG2, and ABCG4) and upregulation of two (ABCC4 and ABCG1) ABC transporters in prostate cancer compared to normal prostate tissue [93]. Hypermethylation of the *ABCB1* promoter has been detected in more than 70% of prostate cancer tissues while it occurred rarely in normal prostate tissues. Neither *ABCB1* gene expression nor *ABCB1* promoter methylation has been found to be correlated with any of the clinical parameters investigated.

The methylation status of a set of genes including *ABCB1* has been determined in serum samples from 192 patients with clinically localized prostate cancer and 18 with hormone refractory metastatic disease in a study by Bastian et al. [160]. Thirty-five serum samples from patients with negative prostate biopsy served as controls. The *ABCB1* promoter was hypermethylated in 38.2% of samples from patients without prostate cancer recurrence and in 16.1% of patients with biochemical recurrence after radical prostatectomy. In the serum from patients with metastatic prostate cancer, the *ABCB1* promoter was hypermethylated in 83.3%, whereas in histologically normal cases hypermethylation of the promoter did not occur.

The findings described above indicate that *ABCB1* promoter hypermethylation is a frequent event in prostate cancer [93,136,137,159,160]. Results mainly refer to CpGs 9–19 and 46–59 (Figure 1). Findings of these studies are consistent although different methodologies have been applied (MSP [93,136,137,159], BS [136,137], *Hpa*II/*Msp*I restriction prior to PCR [160]). The functional role of *ABCB1* promoter methylation in prostate cancer, however, remains to be elucidated.

Liu et al. have identified a prostate carcinoma-initiating stem-like cell subpopulation in the prostate cancer cell line 22Rv1 that is highly prolific, overexpresses ABCG2, and exhibits multidrug resistance [161]. To elucidate the molecular mechanism of drug resistance, the methylation status of the *ABCG2* promoter has been determined. In this subpopulation, the promoter has been found to be hypomethylated. In addition, high levels of histone 3 acetylation and H3K4 trimethylation have been detected.

*ABCA1* promoter hypermethylation and downregulation of *ABCA1* expression has been found to contribute to aberrant accumulation of cholesterol in prostate cancer cell lines [162]. Analyses of prostate cancer and benign prostate tissues have shown that *ABCA1* promoter hypermethylation occurs frequently in prostate cancer but not in benign prostatic tissue. Interestingly, aberrant *ABCA1* promoter methylation was more prevalent in intermediate- and high-grade cancers than in low-grade cancers, suggesting that downregulation of *ABCA1* could play a role in the development and progression of prostate cancer [162].

A study by Devaney et al. aimed to figure out why incidence and mortality rate of prostate cancer are higher in African-American than in Caucasian men [163]. By genome-wide methylation analysis, they have looked for CpGs that were differentially methylated in prostate cancer tissue samples from African-American and Caucasian men. *ABCG5* was one of the genes more frequently hypermethylated in samples from African-American than in samples from Caucasian men. *ABCG5* methylation was inversely correlated with *ABCG5* expression, suggesting that DNA methylation might contribute to the differential aggressiveness of prostate cancer in African-American and Caucasian patients.

### 4.11. Ovarian Cancer

By analyzing three ovarian cancer cell lines, HEY C2, SK-OV-3, and A2780, and three MDR sublines of A2780 selected against cisplatin, A2780/CP70, A2780/MCP2, and A2780/MCP3, ABCA1 has been found to be expressed in A2780/MCP2 and A2780/MCP3 cells but downregulated in A2780 and A2780/CP70 cells [164]. Downregulation of ABCA1 in A2780 and A2780/CP70 cells was associated with methylation of the *ABCA1* promoter. Methylation of the *ABCA1* promoter has neither been observed in primary immortalized normal ovarian surface epithelial (INOSE) nor in primary normal ovarian surface epithelial (NOSE) cells. In the same study, the methylation status of the *ABCA1* promoter has been determined in tissue samples from 76 ovarian cancer patients. Higher *ABCA1* promoter methylation levels have been detected in tumors of higher grade and/or tumors of higher stage. In addition, hypermethylation of the *ABCA1* promoter was associated with prognosis in ovarian cancer patients [164].

Vaclavikova et al. have determined *ABCB1* promoter methylation levels in 61 samples from patients with epithelial ovarian carcinoma [139]. Fifty samples have been collected at the time of surgery (prior to any treatment with chemotherapeutic drugs), eleven samples after neoadjuvant chemotherapy (combination of paclitaxel and platinum derivatives). In 85.2% of the ovarian tumor tissues, the *ABCB1* promoter was significantly higher methylated than in normal ovarian tissues serving as controls. *ABCB1* promoter methylation levels were inversely correlated with *ABCB1* mRNA levels. In the pre-treatment cohort, higher *ABCB1* methylation levels have been observed in tumors at stage I compared to tumors of stages II–IV.

### 4.12. Cervix Cancer

Promoter methylation of *ABCB1*, *ABCC1*, and *ABCG2* has been determined in the cervix cancer line KB-3-1 and two drug-resistant sublines, KBC-1, selected against colchicine and overexpressing ABCB1, and KB-1089, selected against the thiosemicarbazone KP1089 and overexpressing ABCC1 and ABCG2 [142]. Overexpression of ABCB1 in KBC-1 cells has been found to be accompanied by hypomethylation of the *ABCB1* promoter. In KB-1089 cells, overexpression of ABCC1 was due to gene amplification. Overexpression of ABCG2 was mediated neither by gene amplification nor by changes in DNA methylation.

### 4.13. Glioblastoma Multiforme

Oberstadt et al. have assessed the prevalence and prognostic significance of promoter methylation of *ABCB1* and *ABCG2* in glioblastoma multiforme [144]. The promoter methylation status has been determined in tissues from 64 glioblastoma patients, and for a subgroup of 20 patients, mRNA levels have been determined too. The interindividual variability in the promoter methylation status of *ABCB1* (1.3–85.4%) and *ABCG2* (3.6–83.6%) was very high. The *ABCB1* but not the *ABCG2* promoter methylation status was significantly higher in tumor tissues than in healthy brains serving as controls. However, in the cohort of 20 patients, *ABCB1* and *ABCG2* promoter methylation levels were not significantly correlated with *ABCB1* and *ABCG2* mRNA levels.

### 4.14. Leukemia

#### 4.14.1. Acute Leukemia

El-Osta et al. aimed at elucidating the underlying mechanism of *ABCB1* gene regulation in CCRF-CEM, a drug-sensitive T-cell acute lymphoblastic leukemia (T-ALL) cell line, and its subline CEM-A7R, established by selecting CCRF-CEM for resistance against doxorubicin [128,165]. In CCRF-CEM cells, lacking *ABCB1* expression, the *ABCB1* promoter was hypermethylated, whereas in *ABCB1* expressing CEM-A7R cells, the promoter was hypomethylated. By treating the parental cells with 5-aza-dC and trichostatin A and applying chromatin immunoprecipitation, the authors have shown that *ABCB1* promoter methylation was associated with methyl-CpG binding protein 2 (MeCP2) and deacetylated histone. Demethylation and release of MeCP2 from the *ABCB1* promoter resulted in histone acetylation and thus transcriptional activation [128]. The T-ALL cell line CCRF-CEM-Bcl2, stably overexpressing the antiapoptotic protein Bcl2, only moderately expressed *ABCB1* [127]. Exposure of the cells to daunorubicin and etoposide for 8 h or 24 h resulted in a 4–6-fold and ~200 fold upregulation of *ABCB1*, respectively. Upregulation of ABCB1 was associated with changes in histone modification, but alterations in *ABCB1* promoter methylation have not been observed [127].

In the promyelocytic leukemia cell line HL-60, lacking ABCB1 expression, two CpGs (CpG 17 and 37) in the *ABCB1* gene, one upstream and one downstream of the transcription start site, were fully methylated. In the epirubicin resistant subline HL-60/E8, overexpressing ABCB1, these two CpGs were unmethylated [126]. In another study, no differences have been found in the promoter methylation levels of *ABCB1*, *ABCC1*, and *ABCG2* between HL-60 and its drug-resistant sublines HL-60/vinc (vincristine-selected, overexpressing ABCB1 and ABCC1) and HL-60/adr (doxorubicin-selected, overexpressing ABCC1) [142].

Promoter demethylation has been proposed to be the key mechanism underlying overexpression of *ABCG2* in MDR sublines of the T-ALL cell line CCRF-CEM [147]. *ABCG2* promoter demethylation, accompanied by overexpression of *ABCG2*, has been detected after treating CCRF-CEM with the anti-cancer drug sulfasalazine. In addition, the study included T-lymphoblasts and T-cells from four T-ALL patients and four healthy individuals, respectively. *ABCG2* promoter methylation has only been detected in one of the T-ALL patients and none of the healthy patients.

Nakayama et al. have analyzed 42 samples from 31 patients with acute myeloid leukemia (AML) and eight samples from individuals without AML for the *ABCB1* promoter methylation status and *ABCB1* expression [166]. Peripheral blood cells from AML patients showed higher *ABCB1* mRNA levels than peripheral blood cells from healthy individuals. In the AML patient cohort, a statistically significant inverse correlation has been found between *ABCB1* promoter methylation and *ABCB1* gene expression. Moreover, both *ABCB1* promoter methylation and *ABCB1* gene expression levels have been found to change during the clinical course. The highest *ABCB1* promoter methylation and lowest *ABCB1* expression levels have been found at the time of primary diagnosis, the lowest *ABCB1* promoter methylation and highest *ABCB1* expression levels at relapsed state with refractory.

In summary, the findings described above show that alterations in *ABCB1* promoter methylation frequently occur in acute leukemia. However, most studies focused on T-ALL, accounting for about 20% of acute leukemia. Further research is required to elucidate the role of *ABCB1* promoter methylation also in other subtypes of acute leukemia.

#### 4.14.2. Chronic Leukemia

In a study by Moreira et al., gene expression profiles have been compared in the MDR cell lines K-562-Lucena 1 and K-562-FEPS, established by treating chronic myeloid leukemia (CML) K-562 cells with increasing concentrations of vincristine and daunorubicin, respectively [135]. Although the selection process had been different, *ABCB1* was overexpressed in both MDR sublines. *ABCB1* overexpression was associated with hypomethylation of the *ABCB1* promoter. These results suggest a role of *ABCB1* promoter hypomethylation in developing MDR in CML.

In three tumor samples from patients with chronic lymphocytic leukemia (CLL), *ABCB1* promoter methylation levels were inversely correlated with *ABCB1* mRNA levels [165].

Although the studies differed in the CpGs targeted and the methodologies applied (19 CpGs targeted by BS in [135], five CpGs by *Hpa*II/*Msp*I restriction prior to PCR in [165]) (Figure 1), *ABCB1* promoter methylation has been found to be involved in *ABCB1* regulation in both studies.

### 4.15. Multiple Myeloma

Turner et al. have determined the methylation status of the *ABCG2* promoter in multiple myeloma (MM) cell lines and bone marrow aspirated from MM patients [149]. The cell lines included the drug-sensitive MM cell lines NCI-H929 and RPMI 8226, In RPMI 8226MR, a mitoxantrone-resistant subline of RPMI 8226. n RPMI 8226 and its *ABCG2* overexpressing subline RPMI 8226MR, the *ABCG2* promoter was unmethylated. *ABCG2* promoter methylation was inversely correlated with *ABCG2* mRNA levels in NCI-H929 and plasma samples from MM patients. These results suggest that expression of *ABCG2* is regulated, at least in part, by promoter methylation both in cell lines and in bone marrow aspirates from MM patients.

## 5. DNA Methylation of ABC Transporters as Potential Target for Cancer Therapy

Enormous efforts have already been undertaken to overcome MDR in order to enhance the efficacy of anti-cancer drugs [168,169,170]. A widely investigated strategy to overcome MDR is to co-administer anti-cancer drugs with an ABC transporter inhibitor [171]. In order to be applicable, the substance should inhibit the relevant ABC transporter with high potency and specificity, without affecting the pharmacokinetics of the anti-cancer drug [172].

To date, numerous small molecules have been investigated for their potential to reverse MDR by inhibition of ABC transporters [169]. In the beginning, discovery of ABC transporter inhibitors has mainly focused on ABCB1 [173]. Based on their potency and specificity, ABCB1 inhibitors can be classified into three generations [174].

ABCB1 inhibitors of the first generation included calcium channel blockers (e.g., verapamil, nicardipine, nifedipine, diltiazem), calmodulin antagonists (e.g., trifluorperazine, chlorpromazine, trifluopromazine), antibiotics (e.g., erythromycin), the antimalarial drug quinine, and the immunosuppressant cyclosporine A [168]. However, since these molecules had to be applied in very high concentrations, considerable side effects have been observed [170].

In order to increase therapeutic efficacy, ABCB1 inhibitors of the first generation have been modified structurally, leading to ABCB1 inhibitors of the second generation, including dexverapamil, valspodar, and biricodar [174]. These molecules have been found to be more specific, more potent, and less toxic than their analogs of the first generation. However, most ABCB1 inhibitors of the second generation turned out to be substrates of the enzyme cytochrome P4503A4, resulting in unpredictable pharmacokinetic interactions, limiting their application [168].

This problem could be overcome by designing ABCB1 inhibitors of the third generation, e.g., tariquidar, zosuquidar, and laniquidar, lacking affinity for cytochrome P4503A4 [168]. In addition to ABCB1 inhibitors, inhibitors for ABCC1 (e.g., biricodar and ibrutinib) [171,175] and ABCG2 (e.g., imatinib) [171,176,177] have been discovered.

An interesting strategy is the application of anti-cancer drugs and ABC transporter inhibitors by using nanoparticle-based drug delivery systems [178]. Loading both the anti-cancer drug and the ABC transporter inhibitor to a nanocarrier enhances solubility of both substances in aqueous solution, prevents their degradation and enables their controlled and prolonged release [168].

Although clinical trials have shown that co-administration of ABC transporter inhibitors with anti-cancer drugs may result in overall survival of the patients, none of these substances has been approved for clinical use to overcome MDR so far [179]. The main obstacle is that the substances do not specifically inhibit MDR-related ABC transporters but also other ABC transporters of physiological importance.

A further strategy to overcome MDR is to target ABC transporters by using epigenetic modulators. Epigenetic modulators can trigger effects via altering gene expression by epigenetic mechanisms, in particular by targeting DNA methyl transferases (DNMTs) and/or histone modifying enzymes [180]. Increasing evidence suggests that epigenetic modulators have the potential to revert epigenetic aberrations and thus to reprogram neoplastic cells toward a normal state [181]. Since epigenetic aberrations occur early and frequently during carcinogenesis, the use of epigenetic modulators, in combination with other therapies, is considered a promising strategy [181,182,183].

There are two possibilities how an epigenetic modulator can target DNMTs, either by inhibiting DNMTs or by increasing DNMT activity. Epigenetic modulators inhibiting DNMTs show DNA demethylating activity, whereas those increasing DNMT activity lead to DNA hypermethylation [184]. DNMT inhibitors irreversibly inhibit the enzymatic activities of DNMTs and trigger their proteasomal degradation [181]. In addition to synthetic compounds, e.g., 5-azacytidine and decitabine (5-aza-dC), natural compounds, e.g., apigenin, curcumin, and quercetin, have been identified as DNMT inhibitors [185].

Increasing evidence suggests that DNMT inhibitors may have the potential to reactivate genes that have been silenced by DNA hypermethylation, e.g., tumor suppressor genes [186]. Both synthetic and natural DNMT inhibitors have already been tested in vitro and in vivo in combination with other therapies for a variety of cancer types [183,186], including multiple myeloma [184] and other hematological cancers [187]. Currently, only a few epigenetic modulators are used in clinical practice, mainly for treatment of hematological cancers [188]. However, ongoing preclinical and clinical trials investigate their therapeutic potential for solid tumors [189].

As outlined above, overexpression of ABC transporters in MDR cancers is frequently associated with promoter hypomethylation. Thus, MDR in cancer might be overcome by silencing ABC transporters by using epigenetic modulators that show hypermethylating activity by increasing DNMT activity. Two studies have shown that epigenetic modulators increasing DNMT activity offer the possibility to target cancer stem cells [190,191]. In a study by Wang et al., afatinib, a tyrosine kinase inhibitor, has been found to increase DNMT activity, resulting in hypermethylation of the *ABCG2* promoter and lower *ABCG2* mRNA levels in several ABCG2 overexpressing cell lines: MCF7-FLV1000, a flavopiridol-resistant subline of the breast cancer cell line MCF7; S1-M1-80, a mitoxantrone-selected subline of the colon carcinoma cell line S1; CNE-2-s18, a high-metastatic clone of the nasopharyngeal carcinoma cell line CNE2 [190]. By decreasing *ABCG2* expression, cancer stem cell subpopulations could be eliminated in patient-derived leukemia cells. Combining afatinib with the anti-cancer drug topotecan enhanced the efficacy of topotecan in vitro and in vivo.

In a study by Martin et al., melatonin has been found to increase DNMT activity, increase *ABCG2* promoter methylation and downregulate ABCG2 expression in malignant glioma cells as well as in a subpopulation of brain cancer stem cells [191]. Co-incubation of melatonin and either temozolomide, doxorubicin, or mitoxantrone has resulted in increased intracellular concentration and enhanced efficacy of the anti-cancer drug not only in glioma cells but also in brain cancer stem cells.

## 6. Conclusions

To date, most studies investigating DNA methylation of ABC transporters in cancer have focused on *ABCB1* and *ABCG2.* Only a few studies have determined the DNA methylation status of other ABC transporters, including *ABCC1* and ABC transporters playing a role beyond MDR.

The number of DNA methylation data available differs from cancer type to cancer type. Most DNA methylation data have been reported for breast and prostate cancer as well as acute leukemia.

In general, findings on DNA methylation of ABC transporters are not always consistent and sometimes contradictory, even for one and the same cancer type. These differences can, at least in part, be explained by the application of different analytical methodologies and/or the different number and position of CpGs analyzed.

For some cancer types, studies are limited to cancer cell lines and/or MDR cancer cell line models. Thus, there is an urgent need for more studies investigating the clinical relevance of aberrant DNA methylation of ABC transporters in these cancer types.

The *ABCB1* downstream promoter has been found to be commonly hypermethylated in breast and prostate cancer as well as in acute leukemia. MDR cell line models and tumors of the MDR phenotype frequently show *ABCB1* promoter hypomethylation. Data obtained by BS and PSQ hint at aberrant methylation of the whole *ABCB1* promoter region rather than that of single CpGs. *ABCB1* promoter methylation has been found to be inversely correlated with ABCB1 expression at the mRNA and/or protein level in prostate cancer and acute leukemia.

Studies investigating various cell lines, MDR cell line models, and/or clinical samples of different cancer types unambiguously showed that *ABCC1* promoter methylation is not involved in *ABCC1* gene regulation.

Hypermethylation of the *ABCG2* promoter has been reported for colon cancer, multiple myeloma, EBV gastric cancer, and acute leukemia. *ABCG2* promoter hypomethylation has been observed in MDR cell line models of ALL leukemia, ovarian carcinoma, and a stem-like cell subpopulation of prostate carcinoma.

Overcoming MDR by using ABC transporter inhibitors, including substances altering gene expression by targeting DNMTs, is an interesting strategy. To date, limited specificity of these substances hampers their use in routine cancer therapy. However, findings suggest a potential of targeting cancer stem cells by using epigenetic modulators increasing DNMT activity, resulting in *ABCG2* promoter hypermethylation and downregulation of ABCG2.

## Figures and Tables

**Table 1:** Studies investigating DNA methylation of ABC transporters in cancer cell lines.

Cancer	Gene	Method	CpGs	Methylation Status of Cell Line			Study
Type				Low (<25%)	Medium (25%–75%)	High (>75%)	
bladder	*ABCB1*	MSP	5	SCaBER, T24, 5637			[158]
	*ABCC6*		6	SCaBER, 5637	T24		
	*ABCC7*		8		SCaBER, 5637	T24	
breast	*ABCB1*	PSQ	32	BT-474, SK-BR-3,	MCF7, MDA-MB-231, T-47D		[138]
				HMEC (non-cancerous)			
		PSQ	7		ZR-75-1	MCF7, MDA-MB-231	[142]
	*ABCC1*		8	MCF7, MDA-MB-231, ZR-75-1			
	*ABCG2*		8	MCF7, MDA-MB-231, ZR-75-1			
colorectal	*ABCB1*	BS	66	SW620 ^‡^			[127]
	*ABCG2*	BS/MSP	21/2	Caco-2^BS^, COLO 201^BS^, COLO 205^BS^, COLO 320^BS^, DLD-1^BS^, HCT-8^BS^, HCT-15^BS^, HCT 116^BS^, HT-29^BS^, SNU-C1^BS^, SNU-C2A^BS^, SNU-C5^BS^, SNU-61^BS^, SNU-81^BS^, SNU-175^BS^, SNU-283^BS^, SNU-407^BS^, SNU-503^BS^, SNU-769B^BS^, SNU-1033^BS^, SNU-1040^BS^, SNU-1047^BS^, SNU-1197^BS^, SW403^BS^, SW480^BS^, SW1116^BS^, WiDr^BS^	HCT-8^MSP^, HCT-15^MSP^, HCT 116^MSP^, LoVo^BS^, LS 174T ^‡^, NCI-H716 ^‡^, SNU-C2A^MSP^, SNU-C4 ^‡^, SNU-81^MSP^, SNU-175^MSP^, SNU-407^MSP^, SNU-503^MSP^, SNU-769A, SNU-769B^MSP^, SNU-1047^MSP^, SNU-1197^MSP^, SW480^MSP^	Caco-2^MSP^, COLO 201^MSP^, COLO 205^MSP^, COLO 320^MSP^, DLD-1^MSP^, HT-29^MSP^, LoVo^MSP^, SNU-C1^MSP^, SNU-C5^MSP^, SNU-61^MSP^, SNU-1033^MSP^, SW403^MSP^, SW1116^MSP^, WiDr^MSP^	[148]
hepatic	*ABCB6*	BS	82		Hep 3B ^‡^, Hep G2 ^‡^, HuH-6 ^‡^, HuH-7 ^‡^	HLE ^‡^, SK-HEP-1 ^‡^	[156]
leukemia ALL	*ABCB1*	BS	66	CCRF-CEM-Bcl2 (stable Bcl2 overexpression) ^‡^			[127]
	*ABCG2*	COBRA	3	MOLT-4	Jurkat	CCRF-CEM ^‡^	[147]
leukemia CML				K-562			
lung NSCLC	*ABCB1*	PSQ	7	A549, NCI-H520		HCC827, NCI-H1703, SW 1573	[142]
	*ABCC1*		8	A549, HCC827, NCI-H520, NCI-H1703, SW 1573			
	*ABCG2*		8	HCC827, NCI-H520	A549, SW 1573	NCI-H1703	
		MSP	15		NCI-H358, NCI-H441, NCI-H460		[145]
lNung SCLC	*ABCB1*	PSQ	7	DMS 114, GLC-4			[142]
	*ABCC1*		8	DMS 114, GLC-4			
	*ABCG2*		8	DMS 114, GLC-4			
		MSP	15			NCI-H69, PC-6 ^‡^	[145]
myeloma	*ABCG2*	BS	13	RPMI 8226 ^‡^		NCI-H929 ^‡^	[149]
ovarian	*ABCA1*	PSQ	13	HEY C2, SK-OV-3		A2780	[164]
prostate	*ABCA1*	MiGS/BS/MSP	n.a./112/18	DU 145^MiGS^	LNCaP ^‡,MSP^	LNCaP ^‡,BS,MiGS^	[162]
				PrEC (non-cancerous)			
	*ABCB1*	MSP	10	C4-2B, LNCaP	LAPC-4, PC-3	DU 145, VCaP, 22Rv1	[159]
				PrEC, 4ST (non-cancerous)			
		BS/MSP	11/6	LNCaP ^‡^		DU 145 ^‡^, PC-3 ^‡^, 22Rv1 ^‡^	[137]
	*ABCG5*	PSQ	1		LNCaP ^‡^, MDA PCa 2b	DU 145 ^‡^, E006AA, PC-3	[163]
				PNT1A, RWPE-1 (non-cancerous)			
renal	*ABCG2*	BS/*Hpa*II/*Msp*I	66/6	UOK181 ^‡^	UOK121 ^‡^,UOK143 ^‡^		[146]
		450K	10	ACHN, A-498, Caki-1, Caki-2	786-O		[79]

ALL: acute lymphoblastic leukemia, BS: bisulfite sequencing, CML: chronic myeloid leukemia, COBRA: combined bisulfite restriction analysis, CpGs: number of CpGs investigated, MiGS: methyl-CpG-binding domain (MBD) isolated genome sequencing, MSP: methylation-specific PCR, *Hpa*II/*Msp*I: *Hpa*II/*Msp*I restriction prior to PCR, n.a.: not available, NSCLC: non-small cell lung cancer, PSQ: pyrosequencing, SCLC: small cell lung cancer. ^‡^ Cell lines treated with 5-azacytidine or its derivative.

**Table 2:** Studies investigating DNA methylation of ABC transporters in MDR cell line models.

Cancer	Gene	Parental	MDR Cell	Selected by/	Method	CpGs	Association	Study
Type		Cell Line	Line Model	Resistance to			with MDR	
breast	*ABCB1*	MCF7 ^‡^	MCF7/ADR	doxorubicin	BS	66	hypomethylation	[129]
			MCF7/TXT ^‡^	docetaxel	BS	66	hypermethylation	[130]
		MCF7 ^‡^	MCF7/DOX-2	doxorubicin	BS	66	none	[131]
			MCF7/EPI	epirubicin	BS	66	hypomethylation	[131]
			MCF7/TAX-2	paclitaxel	BS/PSQ	66/18	hypomethylation	[131]
			MCF7/R	doxorubicin	*Hpa*II/*Msp*I	4	hypomethylation	[151]
	*ABCG2*	MCF7	MCF7/MR	mitoxantrone	COBRA	3	none	[147]
			MCF7-FLV1000	flavopiridol	COBRA	3	none	
cervical	*ABCB1*	KB-3-1	KBC-1	colchicine	PSQ	7	hypomethylation	[142]
			KB-1089	gallium complex	PSQ	7	hypomethylation	
	*ABCC1*	KB-3-1	KBC-1	colchicine	PSQ	8	none	[142]
			KB-1089	gallium complex	PSQ	8	none	
	*ABCG2*	KB-3-1	KBC-1	colchicine	PSQ	8	none	[142]
			KB-1089	gallium complex	PSQ	8	none	
colorectal	*ABCB1*	SW480	SW480/tria	triapine	PSQ	7	hypomethylation	[143]
esophageal	*ABCB1*	TE-1 ^‡^	RTE-1D	docetaxel	BS	56	hypomethylation	[133]
			RTE-1P	paclitaxel	BS	56	hypomethylation	
leukemia ALL	*ABCB1*	CCRF-CEM ^‡^	CEM-A7, -A7R	doxorubicin	*Hpa*II/*Msp*I	5	hypomethylation	[165]
		CCRF-CEM ^‡^	CEM-A7R ^‡^	doxorubicin	BS	66	hypomethylation	[128]
			CCRF-CEM/SSZ	sulfasalazine	COBRA	2	none	[147]
	*ABCG2*	CCRF-CEM ^‡^	CCRF-CEM/SSZ ^‡^	sulfasalazine	BS/COBRA	38/3	hypomethylation	[147]
leukemiaAML	*ABCB1*	HL-60	HL-60/E8	epirubicin	BS	66	hypomethylation	[126]
			HL-60/adr	doxorubicin	PSQ	7	none	[142]
			HL-60/vinc	vincristine	PSQ	7	none	[142]
	*ABCC1*	HL-60	HL-60/adr	doxorubicin	PSQ	8	none	[142]
			HL-60/vinc	vincristine	PSQ	8	none	
	*ABCG2*	HL-60	HL-60/adr	doxorubicin	PSQ	8	none	[142]
			HL-60/vinc	vincristine	PSQ	8	none	
leukemiaCML	*ABCB1*	K-562	K-562-Lucena 1	vincristine	BS	19	hypomethylation	[135]
			K-562-FEPS	daunorubicin	BS	19	hypomethylation	[135]
			K-562/ADR	doxorubicin	BS	22	hypomethylation	[133]
lung NSCLC	*ABCB1*	A549 ^‡^	A549/DDP	cisplatin	BS	66	hypermethylation	[132]
		SW 1573	SW 1573/2R120, 2R160	doxorubicin	PSQ	7	hypomethylation	[142]
	*ABCC1*	SW 1573	SW 1573/2R120, 2R160	doxorubicin	PSQ	8	none	[142]
	*ABCG2*	SW 1573	SW 1573/2R120, 2R160	doxorubicin	PSQ	8	none	[142]
		A549	A549/K1.5	triazoloacridone	COBRA	3	none	[147]
Nlung SCLC	*ABCB1*	GLC-4	GLC-4/adr, rev	doxorubicin	PSQ	7	hypermethylation	[142]
	*ABCC1*	GLC-4	GLC-4/adr, rev	doxorubicin	PSQ	8	none	[142]
	*ABCG2*	GLC-4	GLC-4/adr, rev	doxorubicin	PSQ	8	hypermethylation	[142]
		PC-6 ^‡^	PC-6/SN2-5H	irinotecan	BS/MSP	91/15	hypomethylation	[145]
myeloma	*ABCG2*	RPMI 8226 ^‡^	RPMI 8226MR	mitoxantrone	BS	13	none	[149]
ovarian	*ABCA1*	A2780	A2780/CP70 ^‡^	cisplatin	PSQ	13	none	[164]
			A2780/MCP2, MCP3	cisplatin	PSQ	13	hypomethylation	
	*ABCG2*	IGROV-1 ^‡^	IGROV-1-MX3 ^‡^	mitoxantrone	COBRA	3	hypomethylation	[147]
			IGROV-1/T8 ^‡^	topotecan	BS/COBRA	38/3	hypomethylation	
pancreatic	*ABCB1*	SW1990	SW1990/GZ	gemcitabine	BS	25	none	[134]
	*ABCC1*		SW1990/GZ	gemcitabine	BS	23	none	
	*ABCG2*		SW1990/GZ	gemcitabine	BS	25	none	
prostate	*ABCG2*	22Rv1 ^‡^	22Rv1CD117+ABCG2+	(subpopulation)	MSP	9	hypomethylation	[161]

ALL: acute lymphoblastic leukemia, AML: acute myeloid leukemia, BS: bisulfite sequencing, CML: chronic myeloid leukemia, COBRA: combined bisulfite restriction analysis, CpGs: number of CpGs investigated, MDR: multidrug resistance, MSP: methylation-specific PCR, *Hpa*II/*Msp*I: *Hpa*II/*Msp*I restriction prior to PCR, NSCLC: non-small cell lung cancer, PSQ: pyrosequencing, SCLC: small cell lung cancer. ^‡^ Cell lines treated with 5-azacytidine or its derivative.

**Table 3:** Studies investigating DNA methylation of ABC transporters in clinical samples.

Cancer Type	Gene	and number of patients	Method	CpGs	Association with	Study
bladder	*ABCB1*	untreated primary tumor = 23 (2 ^§^,1 ^§^), relapse = 16 (2 ^§^,1 ^§^), residual = 12 (1 ^§^,1 ^§^)	*Hpa*II/*Msp*I	4	recurrence: hypomethylation resistance: hypomethylation	[157]
	*ABCC6*	primary tumor = 15, control = 3; urine from patients with primary tumor = 99, relapse = 33, from controls = 36	MSP	6	cancer: hypermethylation recurrence: none	[158]
	*ABCC7*	primary tumor = 15, control = 3; urine from patients with primary tumor = 99, relapse = 33, from controls = 36	MSP	8	cancer: hypermethylation recurrence: none	
brain GBM	*ABCB1*	untreated primary tumor = 64 (17 ^§^), relapse = 17 ^§^, control = 7	PSQ	2	cancer: hypermethylation recurrence: differently methylated	[144]
	*ABCG2*	untreated primary tumor = 64 (17 ^§^), relapse = 17 ^§^, control = 7	PSQ	3	cancer: none recurrence: none	
breast	*ABCB1*	untreated primary tumor = 100 (15 ^§^), distant = 15 ^§^; blood from patients with untreated primary tumor = 100 ^§^, from controls = 30	MSP	6	cancer: hypomethylation progression: hypomethylation	[152]
		primary tumor = 163, untreated primary tumor = 75 ^‡^ (68 ^§^), treated primary tumor = 68 ^§^, control = 6 ^‡^	PSQ	39	cancer: hypermethylation ^$^ resistance: hypomethylation	[138]
		primary tumor = 89 (71 ^†^), control = 28	PSQ	20	cancer: hypermethylation ^$^ progression: hypomethylation	[141]
		untreated primary tumor = 75 ^‡,^ treated primary tumor = 35 (34 ^Ɨ^), primary tumor = 128 (71 ^†^), control = 6 ^‡^	PSQ	20	cancer: hypermethylation ^$^ progression: hypermethylation	[140]
		untreated primary tumor = 83 (6 ^§^), adjacent = 6 ^§^, treated primary tumor = 112 (34 ^Ɨ^)	PSQ/MS-HRM	39/49	cancer: none resistance: none	[139]
		untreated primary tumor = 16 ^§^, adjacent = 16 ^§^, distant = 16 ^§^, control = 4	PSQ	7	cancer: hypermethylation	[142]
	*ABCC1*	untreated primary tumor = 16 ^§^, adjacent = 16 ^§^, distant = 16 ^§^, control = 4	PSQ	8	cancer: none	
	*ABCG2*	untreated primary tumor = 16 ^§^, adjacent = 16 ^§^, distant = 16 ^§^, control = 4	PSQ	8	cancer: none	
esophageal	*ABCD1*	TCGA primary tumor = 96, TCGA control = 3	450K	18	cancer: hypomethylation	[153]
gastric	*ABCB1*	primary = 83 ^§^ (77 *H. pylori*+), adjacent = 83 ^§^	MSP	7	cancer: hypermethylation	[154]
	*ABCG2*	lymph node = 1 (EBV), TCGA primary tumor = 4 (non-EBV), TCGA control = 2	Epic/450K	9	EBV: methylation	[155]
hepatic	*ABCB6*	untreated primary tumor = 81 (53 HCV, 28 non-HCV)	MSP	9	recurrence in HCV: hypomethylation	[156]
leukemia AML	*ABCB1*	blood (MNC), BMA (MNC) from patients having primary tumor with remission = 13, resistance = 10, relapse = 8 (2 remission, 6 resistance), from controls = 8	*Hpa*II/*Msp*I	4	cancer: hypermethylation resistance: hypomethylation relapse: hypomethylation	[166]
leukemiaCLL		blood (B-cells, C5/19+) from patients with primary tumor = 3 (2 MDR)	*Hpa*II/*Msp*I	5	resistance: hypomethylation	[165]
leukemiaALL	*ABCG2*	blood (T-cells) from patients with primary tumor = 4, from controls = 4	COBRA	3	cancer: none	[147]
lung NSCLC	*ABCB1*	untreated primary tumor = 20 ^§^, adjacent = 20 ^§^	BS	66	cancer: hypermethylation	[132]
		primary tumor = 36, control = 10	MSP	4	cancer: hypomethylation progression: none	[150]
myeloma	*ABCG2*	BMA (CD138+) from patients with primary tumor = 8	MSP	2	cancer: methylation	[149]
ovarian	*ABCA1*	untreated primary tumor = 76 (49 high, 27 low stage), control = 8	PSQ	13	cancer: hypermethylation progression: hypermethylation	[164]
	*ABCB1*	untreated primary tumor = 50, treated primary tumor = 11, control = 11	PSQ	39	cancer: hypermethylation progression: hypomethylation resistance: hypomethylation	[139]
prostate	*ABCA1*	untreated primary tumor = 30 (23 high, 7 low stage), control = 9	MSP	4	progression: hypermethylation	[162]
	*ABCB1*	untreated primary tumor = 78, control = 19	MSP	4	cancer: hypermethylation progression: none	[93]
		untreated primary tumor = 73 (12 ^§^), metastasis = 36, adjacent = 12 ^§^, control = 13	MSP	10	cancer: hypermethylation metastatic site: none	[159]
		untreated primary tumor = 121, pre-malignant = 37, control = 36	MSP	6	cancer: hypermethylation progression: hypermethylation	[137]
		untreated primary tumor = 177 (80 high, 97 low stage), control = 79	BS/MSP	11/6	cancer: hypermethylation progression: hypermethylation	[136]
		blood from patients with untreated primary tumor = 192, refractory metastases = 18, from controls = 35	*Hpa*II/*Msp*I	2	cancer: hypermethylation metastasis: hypermethylation	[160]
	*ABCG5*	untreated primary tumor = 62 ^§^ (29 AA, 33 Cau), control = 71 (62 ^§^)	450K/PSQ	18/1	AA cancer: hypermethylation	[163]
renal	*ABCG2*	untreated primary tumor = 16, TCGA untreated primary tumor = 274 (143 ^§^), TCGA adjacent = 143 ^§^	450K	10	cancer: none	[79]

AA: African-American, ALL: acute lymphoblastic leukemia, AML: acute myeloid leukemia, BMA: bone marrow aspirate, BS: bisulfite sequencing, Cau: Caucasian, CLL: chronic lymphoblastic leukemia, CML: chronic myeloid leukemia, COBRA: combined bisulfite restriction analysis, CpGs: number of CpGs investigated, EBV: Epstein–Barr virus, GBM: glioblastoma multiforme, HCV: hepatitis C virus, MDR: multidrug resistance, MNC: mononuclear cells, MSP: methylation-specific PCR, *Hpa*II/*Msp*I: *Hpa*II/*Msp*I restriction prior to PCR, NSCLC: non-small cell lung cancer, PSQ: pyrosequencing, TCGA: The Cancer Genome Atlas. ^§^ paired samples derived from same patients analyzed within the respective study. ^$^ except in molecular subtype basal-like/triple negative, normal-like. ^‡, †, Ɨ^ same samples analyzed in different studies for the same gene.
